# Xeroderma Pigmentosum: A Genetic Condition Skin Cancer Correlated—A Systematic Review

**DOI:** 10.1155/2022/8549532

**Published:** 2022-07-18

**Authors:** Tito Brambullo, Michele Rosario Colonna, Vincenzo Vindigni, Stefano Piaserico, Giuseppe Masciopinto, Mariarosaria Galeano, Alfio Luca Costa, Franco Bassetto

**Affiliations:** ^1^Plastic and Reconstructive Surgery, Neuroscience Department, University of Padua, Via Giustiniani 2, Padova 35128, Italy; ^2^Department of Human Pathology, University of Messina, Via Consolare Valeria 1, Azienda Ospedaliera Universitaria Policlinico “G. Martino” 98125 Messina, Italy; ^3^Dermatology Unit, Department of Medicine-DIMED, University of Padova, Via Gallucci 4, IT-35121 Padova, Italy

## Abstract

**Background:**

Xeroderma pigmentosum (XP) is a rare autosomal recessive disorder of UV radiation-induced damage repair that is characterized by photosensitivity and a propensity for developing, among many others, skin cancers at an early age. This systematic review focused on the correlation between the clinical, pathological, and genetic aspects of XP and skin cancer.

**Methods:**

A systematic review was conducted through a literature search of online databases PubMed, Cochrane Library, SciELO, and Google Scholar. Search terms were “Xeroderma pigmentosum”, “XP”, “XPC”, “Nucleotide excision repair”, “NER”, “POLH”, “Dry pigmented skin”, and “UV sensitive syndrome” meshed with the terms “Skin cancer”, “Melanoma”, and “NMSC”.

**Results:**

After 504 abstracts screening, 13 full-text articles were assessed for eligibility, and 3 of them were excluded. Ten articles were selected for qualitative assessment.

**Conclusions:**

Patients with XP usually suffer shorter lives due to skin cancer and neurodegenerative disease. Deletion/alteration of a distinct gene allele can produce different types of cancer. The XPC and XP-E variants are more likely to have skin cancer than patients in other complement groups, and the most common cause of death for these patients is skin cancer (metastatic melanoma or invasive SCC). Still, aggressive preventative measures to minimize UV radiation exposure can retard the course of the disease and improve the quality of life.

## 1. Introduction

Xeroderma pigmentosum (XP) is a rare autosomal recessive disorder characterized by a deficiency in nucleotide excision repair (NER) caused by single-nucleotide mutations [1].

Sunlight exposure is usually the trigger for UV-induced damage to DNA, which normally is repaired through the NER pathway, consisting in more than 30 proteins responsible for DNA damage recognition, incision, ligation, and resynthesis [2].

XP incidence rate is estimated to be 1 in 250,000 to 1 in 1,000,000 in North America and Europe [3–4]. Still, it increases in countries where consanguinity is more common, such as Japan (1 in 20,000) [5], India, the Middle East, and Northern Africa (1 in 10,000-30,0000) [6].

XP can be further subdivided into seven distinct subgroups, known as complementation groups, XP-A through to XP-G, and a XP-variant (XP-V), usually considered a milder variant form [7].

Each complementation group refers to the presence of a causative mutation in one of the seven XP genes involved in NER [8] or POLH (XP-V) [9] involved in translation synthesis.

One more affected gene can be ERCC1, but its mutation is rare.

The abovementioned pathways with altered gene expression contain oncogenic-related functions such as perturbation of cell cycle, apoptosis, proliferation, and differentiation.

Several types of malignancy have been correlated with XP syndrome: gastric, breast, bladder, colorectal, lung, endometrial, brain, head and neck, prostate and melanoma, and nonmelanoma skin cancers (NMSC) [10].

In particular, the deficiency in functional NER results in up to 2000 times greater susceptibility to melanoma and a 10,000-fold increase in basal cell carcinomas (BCCs) and squamous cell carcinomas (SCCs) [11].

Besides skin cancers and internal tumors, 20-30% of XP patients suffer from progressive neurological degeneration [12–13].

Neurological forms of XP can be classified into three distinct groups: XP neurologic disease, XP with trichothiodystrophy (XP-TTD), and xeroderma pigmentosum-Cockayne syndrome complex (XP-CS) [14].

The location of the affected gene in the NER pathway does not confer the severity of DNA repair deficiency and subsequent clinical manifestation of the disease. Therefore, accurate diagnosis of XP, CS, and TTD currently relies on clinical assessment and identification of mutations in the NER genes [15].

## 2. Nucleotide Excision Repair

**Figure 1:**
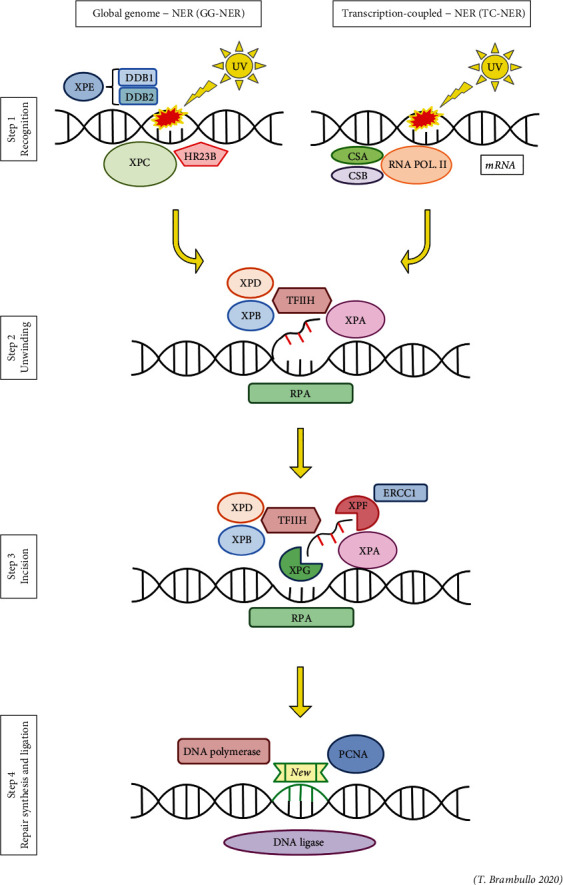
The nucleotide excision repair (NER) pathway. Step 1: DNA damage recognition through two different ways: global genome (GG-NER) or transcription-coupled (TC-NER). Step 2: DNA unwinding. Step 3: DNA damage excision. Step 4: missing strand synthesis and ligation.

The nucleotide excision repair (NER) pathway can start with DNA damage recognition in two different ways: global genome (GG) or transcription-coupled (TC) (Figure 1).

Via global genome, the damage detection occurs across the remainder of the genome and involves XPC and the damage DNA-binding protein 1 and 2 (DDB1/DDB2) complex (step 1).

Otherwise, the transcription-coupled system detects DNA damage during transcription when RNA polymerase II stalls at a site of damage recruiting Cockayne syndrome group A and B proteins (CSA, CSB).

After the DNA damage is detected via GG or TC, the remainders of the XP proteins are involved in DNA unwinding (XPA, XPB, and XPD—step 2) and excision of the damage (XPF, ERCC1, and XPG—step 3) [15].

Next, the DNA polymerase delta (Pol *δ*) initiates missing strand synthesis recruiting the proliferating cell nuclear antigen (PCNA), the cofactor for DNA clamping, while the DNA ligase connects the two strands by forming a bond between the phosphate group of one strand and the deoxyribose group on another, so maintaining DNA integrity during the repair process (step 4).

## 3. Methods

### 3.1. Aim of the Study

The focus of the present study is to determine which subgroups of XP have the highest correlation with the development of skin cancers and outline the clinical features of the syndrome and the best treatment strategies.

### 3.2. Study Design

A systematic review was conducted through a literature search of online databases PubMed, Cochrane Library, SciELO, and Google Scholar between August and September 2020.

Search terms were “Xeroderma pigmentosum”, “XP”, “XPC”, “Nucleotide excision repair”, “NER”, “POLH”, “Dry pigmented skin”, and “UV sensitive syndrome” meshed with the terms “Skin cancer”, “Melanoma”, and “NMSC” using the Boolean terms “AND” and “OR” to better improve the research.

**Figure 2:**
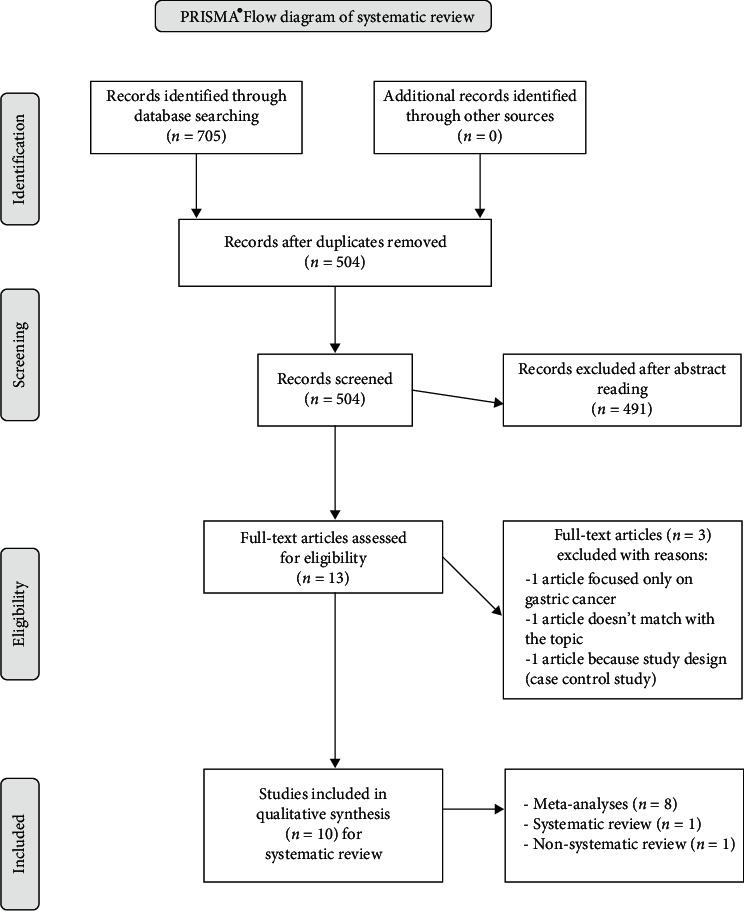
PRISMA diagram. Summary of the records identified through database searching.

A PRISMA® flow diagram [16] summarizes the records identified through database searching and included in the final box the ones in the systematic review (Figure 2).

### 3.3. Search Criteria

Database searching filters were high evidence studies (meta-analysis, review, randomized controlled trial, clinical trial, cohort study, and consensus conference). Low-evidence studies (case-control study, small case series, and case report) were excluded.

All the publications have been edited English language over the last 10 years and strictly fit the topic (correlation between XP and skin cancer).

### 3.4. Evidence Evaluation

Due to the heterogeneity of the records selected in the review, the GRADE® rating [17] was applied to the results to present summaries of evidence and provide a systematic approach and quality assessment.

## 4. Results

Articles selected [2, 7, 10, 14, 18–23] for qualitative assessment are presented in Table 1. After 504 abstracts screening, 13 full-text articles were assessed for eligibility, and 3 of them were excluded: one article was focused only on the association of XP with gastric cancer, one did not fit the topic and represented a limit of the online database search, one article had a control study design, so was excluded having low evidence level.

The remaining articles were 8 meta-analyses, 7 inherent to the association between selected XP completion groups and all cancer types, one focused only on melanoma cancer, and 2 reviews: one systematic and the other nonsystematic.

## 5. Discussion

Xeroderma pigmentosum is a genetic alteration that predisposes to develop a large spectrum of solid cancers, among which are skin tumors [24, 25].

Distinct gene allele deletion/alteration can produce different cancers, as genetic research confirmed with several publications [26–28].

However, skin tumors (melanoma, basal cell carcinoma, and squamous cell carcinoma) represent only a small part of overall malignancies associated with the XP syndrome. Few studies were conducted to analyze which type of complementation group is more likely to generate them.

An additional problem lies in the ascription of the correct etiology because different complementation groups can produce similar clinical features [29], rendering the syndrome much more confusing and more challenging to identify cancer origin.

As shown in Table 1, only a few reports suggest some correlation between a single XP complementation group and skin cancer. Besides the intrinsic bias of the different studies, a great limitation for adequate risk assessment relies on the small population [30, 31] enrolled and the selected predisposition of some populations due to ethnicity [32–34].

Zhao et al. [10] and He et al. [21], in their meta-analysis, attest that allele mutation of the XPG complementation group, rs17655 and Asp1104His, may increase the overall cancer risk but did not correlate these polymorphisms to a specific risk of cutaneous malignancies.

The former encompasses only 8 studies on skin cancer, and the second 10, and both of them refer to studies that used selected hospital patients without organic cancer as the reference group, so including in the control group people with different risks of developing cancer (age, sex, comorbidities, etc.); thus, this method could lead to a misclassification bias.

Even Han et al. [22] failed to prove any association between selected XPG polymorphisms and skin cancer. Still, sample sizes and the control group of some original studies (enrolling other patients without malignant tumors) may be a source of selection bias.

Natale and Raquer [14], in a systematic review, report an estimation of risk for NMSC 10,000 times greater in XP patients than in the unaffected population and >2,000 times greater for melanoma [11].

This affirmation refers to Bradford et al.'s [11] retrospective follow-up study of 106 XP patients, of which only 78 were non-Hispanic whites (only partially obviating the ethnicity bias, n.d.r.).

In that study, a follow-up was obtained via telephone; clinical data were collected using a standard questionnaire completed at the time of the initial visit and medical records, including pathology reports of skin and other cancers and other sources.

The expected number of deaths was calculated by applying the US mortality rates to the appropriate person-time accrued by XP survivors in the cohort.

The conclusion was that many patients in complementation groups XPC, XP-E, and variant never burned but were more likely to have skin cancer than patients in complementation groups XP-A, XP-B, XP-D, and XP-G, and that the most common cause of death was skin cancer (metastatic melanoma or invasive SCC).

A number of biases affect this study, and the absence of controlling a group leads to rating the overall evidence quality as very low. Still, it represents a valuable basis for further research with a more solid study design.

Natale and Raquer [14] do not provide a clear stratification of studies included in the review; only 5 of 33 were mentioned reporting correlation with skin cancer, but the data presentation form does not consent to correlate the risk of cancer to a specific XP or XP-CS complementation group nor to have a comprehensive survey on the entire data collection.

Therefore, the evidence quality of this study is consequently rated as very low.

XPC complementation group seems to correlate with skin cancer. Zebian et al. [7] report 6 studies on skin cancer in humans and 7 on lab rats, arguing on XPC role as a sensor of helical distortions in the GG-NER pathway, and as XPC^−/−^ mice show a high predisposition to UV radiation-induced skin tumor.

These data suggest a tumor suppressor role of XPC during cancer progression.

This study is a nonsystematic review and carries data selection bias that affects its evidence quality.

With specific regard to melanoma occurrence, Jiang et al. [23] investigated the correlation with a single XPC polymorphism, but the study failed to prove a strong association and was based on unadjusted estimates for age, gender, and nationality, so compromising the quality of evidence.

Ding et al. [2], in a meta-analysis of 36 studies, found a reduced risk of BCC and SCC in patients affected by XPA-4G>A polymorphism, but nondifferential misclassification bias is somehow possible due to the inclusion in a control group of hospital patients without organic cancer.

Globally, the study failed to prove any strong association between a single XP complementation group and skin cancer, but highlighted a greater tendency of some of them to develop such type of malignancy.

Instead, the overall increased risk of cancer can be confirmed for all patients affected by xeroderma pigmentosum.

## 6. Clinical Features of XP

### 6.1. Common History

Since childhood, XP patients start experimenting in their life with multiple occurrences of skin cancers; most of them are epitheliomas, usually basal cell carcinoma and squamous cell carcinoma, but even more rare as Merkel cell carcinoma.

Along with them, several precancerous neoplasms, such as actinic keratosis and keratoacanthoma, may arise in any region of the body surface, with the highest predilection for sun-exposed areas.

Malignant melanoma is a common finding in xeroderma and often determines the overall treatment given its life-threatening capacity.

Thus, the clinical feature of an adult XP patient can be characterized by the presence of simultaneous skin cancers in a framework of altered skin and scars of previous surgery.

Frequent biopsies and excisional therapies can progressively lead to amputation in areas of high aesthetic importance, such as the ear, nose, lip, and eyelid.

Besides the consequent impairment of function, the loss of structures crucial for self-esteem can drive the patient to depression and reduced social life.

The scenario seems much more devastating if we consider that in the early part of their lives, XP patients experience a normal social life and do not appreciate any future symptoms of their genetic disease.

### 6.2. Clinical Case

**Figure 3:**
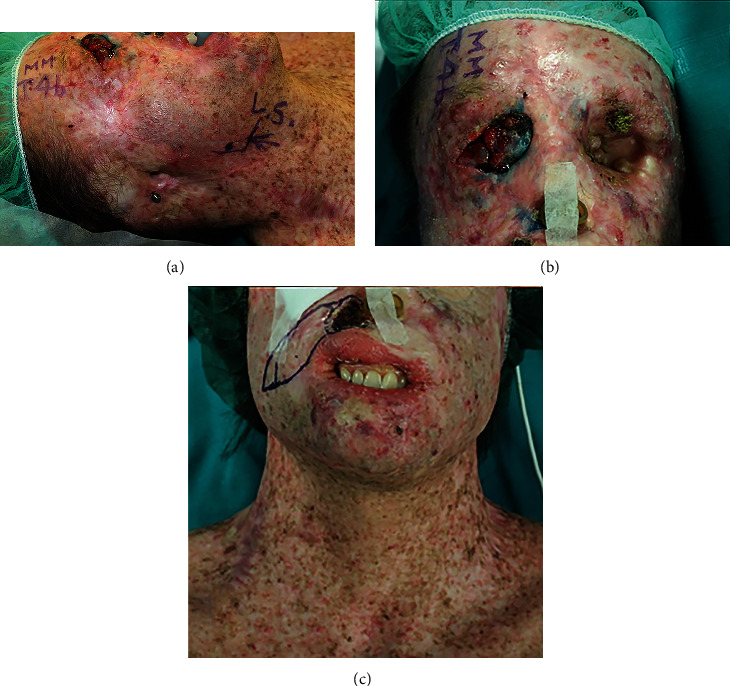
Clinical case of a 28-year-old male patient affected by xeroderma pigmentosum. Absence of the right auricle with bone anchor for cosmetic prosthesis, planning for sentinel lymph node biopsy for pT4 malignant melanoma of the right superior eyelid (a). Superior third of the face just before the second (right) exenteratio orbitae, nasal splint for preventing collapsing of nostril residuals (b). Superior lip retraction, planning of local skin flap for BCC of the right nasolabial fold, and note the surgical scars of previous surgeries at the cheek, jawline, and cervical levels (c).

In Figure 3, we present the clinical case of a 28-year-old male patient affected by xeroderma pigmentosus-complementation group C (XPC) that has been treated for several NMSC since his teen years.

**Figure 4:**
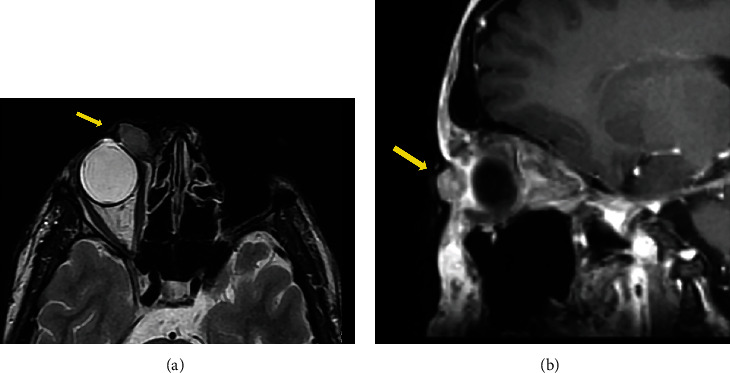
MR scan C+ delayed of orbits. A solid neoplastic tissue of the medial cantus invading the right orbital space, malignant melanoma pT4b (yellow arrows), and axial (a) and sagittal (b) views.

Because of several surgical procedures, he has lost both of the auricles, the entire external nose, and part of the upper lip, and he went through a double exenteratio orbitae due to cancer invading orbital spaces (Figure 4).

The surrounding skin of the head and neck areas harbors several pigmented lesions, most of which are acquired during growing.

Furthermore, he sustained several biopsies and incisions at the trunk and limb levels.

The patient's dermis and subcutaneous fat layer have lost elasticity and softness, and the overall skin envelope appears under tension and somehow retracted.

Even when a single lesion is well defined on the skin surface, free surgical margins are difficult to get because of the frequent presence of adjacent other lesions that may result in confounding findings for both surgeon and pathologist.

Contrary to usual, in XP patients, even small excision often requires accurate reconstructive planning because of an absence of skin pliability and consequent failure of conventional skin border approximation and suture.

The plastic surgeon has to master all the tools of reconstructive armamentarium to deal with this challenging disease; nevertheless, aesthetic and functional outcomes are invariably affected, so the surgical treatment is far from satisfactory.

For all the reasons above, other clinical figures such as dermatologists, oncologists, and radiotherapists play a capital role in early diagnosis and alternative therapies, in the effort to limit as much as possible the need for wide and disfiguring resections.

## 7. Therapy

### 7.1. Surgery and Alternative Procedures

Radical resection with free margins is the standard treatment for primitive malignant melanoma.

The lateral extension of skin incision varies with Breslow's parameter (melanoma thickness-the measure of vertical distance of the lesion, from the more superficial edge to the deepest cell nest, expressed in mm), while the deep margin usually consists of the deep muscle fascia [35].

Usually, after removal of the nevus, wide excision is planned, together with the biopsy of the sentinel lymph node, a standard practice that has proved to be predictive of distant metastasis in the thin melanoma (less than 4 mm in thickness) [36].

The surgical procedure is always preceded by instrumental staging with a TC scan for the thorax and abdomen, MR or CT scan for the brain, and ultrasounds for regional lymph nodes, to exclude distant metastasis [37], which may indicate an immediate systemic therapy [38].

Nonmelanoma skin cancers, as other solid tumors, need surgical resection with free margins because 90% of lesions are located in sun-exposed sites such as the face or neck, which can result in a significant disfigurement [39–41].

Unlike melanoma, NMSC often requires reconstruction techniques because the more aggressive local behavior leads the surgeon to wider resections to obtain free margins and low recurrence rates.

Moreover, in many cases, many lesions in the same area make conventional resections impractical [42].

When small in dimensions and still not invasive, they are successfully treated with therapies alternative to the ablative procedure [43].

Photodynamic therapy (PDT) is widely applied in dermatology to treat nonmelanoma skin cancer premalignant and malignant lesions (actinic keratosis, basal cell carcinoma, and in situ squamous cell carcinoma).

In PDT, the interaction of a photosensitizer (PS), light, and oxygen leads to the formation of reactive oxygen species (ROS) that are extremely harmful to cell viability; they can activate irreversible apoptotic and necrotic cell death mechanisms with consequent tumor cell eradication.

Other topic therapies comprehend imiquimod as monotherapy for superficial BCC and fluorouracil as monotherapy for superficial BCC and SCC in situ [44].

Their use has to be limited to patients with small tumors in low-risk locations who are not candidates for standard ablative treatment.

Limitations of the aforesaid therapies include local adverse effects, such as skin flushing and inflammation often associated with pain and discomfort, lower clearance rates, dependence on patient compliance and adherence to treatment, and higher costs for prolonged treatments.

### 7.2. Pharmacotherapy

#### 7.2.1. Melanoma

Several therapies have been developed to stimulate an antitumor immune system response toward melanoma [45].

The first immune therapy developed for metastatic melanoma was the interleukin-2 (IL2), characterized by a partial rate of effectiveness.

Toxicity is generally cumulative, becoming worse and harder to manage with the cycle progression; thus, the treatment may be stopped earlier if adverse events make further administration intolerable for the patient [46].

IL2 induces reliable and durable antitumor responses and is likely curative in a small subset of patients [47].

Recently, immune checkpoint inhibitors (CPI) have proved to be effective treatments for metastatic melanoma [48].

The immune checkpoint inhibitor drugs approved for melanoma treatment are PD-1 inhibitors pembrolizumab and nivolumab, PD-L1 inhibitor atezolizumab, and CTLA-4 inhibitor ipilimumab.

Immunotherapy also includes oncolytic virus therapy (talimogene laherparepvec T-VEC) [49] and imiquimod cream [50].

Target therapy has become an appropriate first-line adjuvant treatment of mutant-advanced cutaneous melanoma patients [51]:

BRAF inhibitors (vemurafenib, dabrafenib, and encorafenib), MEK inhibitors (trametinib, cobimetinib, and binimetinib), and drugs that target cells with C-KIT gene changes (imatinib and nilotinib).

Finally, treating patients with systemic melanoma should include chemotherapy, although its role remains relegated to palliative care [52].

Several chemotherapeutic agents have been successfully tested: dacarbazine, temozolomide, nab-paclitaxel, paclitaxel, cisplatin, and carboplatin, but the high rates of side effects, systemic toxicity, and a not definitely curative effect determine their use as a second-line treatment.

A particular variant of chemotherapy is the isolated limb perfusion (ILP) [53] which consists of the perfusion of a limb isolated from the systemic circulation and connected to an extracorporeal system.

Then, the chemotherapy drugs, melphalan and tumor necrosis factor (TNF), are administered to the patient through the perfusion circuit, minimizing the toxicity related to systemic chemotherapy [54].

Isolated limb perfusion is a treatment modality used for unresectable melanoma in-transit metastases, and ILP lengthens the limb recurrence-free interval and decreases the number of lesions per recurrence significantly in patients with repeatedly recurrent limb melanoma [55].

With time, the utilization of ILP has decreased for melanoma but remains an important option to consider as a regional disease control alternative to limb amputation.

#### 7.2.2. Squamous Cell Carcinoma

The first treatment options for cutaneous squamous cell carcinoma included conventional chemotherapy, epidermal growth factor receptor- (EGFR-) targeted therapy, and interferon [56].

Today, the immune checkpoint inhibitor cemiplimab, a humanized IgG4 antibody directed against the programmed cell death-1 (PD-1) receptor, represents the agent of choice.

The role of CPI in XP patients is unclear: the high tumor mutational burden suggests cemiplimab could be useful and isolated case reports confirmed effectiveness [57].

However, clinical observation can be sometimes controversial, while metastatic disease responds to CPI, and new SCC does occur [58].

Currently, the CPI is used for treating patients with unresectable, locally advanced, or metastatic SCC.

#### 7.2.3. Basal Cell Carcinoma

A recent definition of the role of hedgehog signaling in the pathogenesis of basal cell carcinoma has facilitated the development of treatment options with improved clinical outcomes.

In humans, the hedgehog signaling pathway regulates development, cell proliferation, and tissue repair.

Dysregulated hedgehog pathway components (e.g., smoothened (Smo) and patched-1) lead to constant activation and consequently act as potent oncogenic driver signals [59].

Thus, inhibitors block the hedgehog signaling either at the level of Smo (i.e., vismodegib, sonidegib, patidegib, and itraconazole) or via an unknown mode of action (arsenic trioxide) can inhibit basal cell carcinoma occurrence.

The most common treatment-emergent adverse events associated with approved hedgehog inhibitors include muscle spasms, dysgeusia, and alopecia.

BCC patients treated with the hedgehog pathway inhibitors frequently experienced relapse during a follow-up after drug discontinuation, but an objective response to vismodegib second administration is present in about 50% of them [60].

In Europe, the use of vismodegib and sonidegib has been approved for the treatment of patients with unresectable, locally advanced, or metastatic BCC to date.

#### 7.2.4. Radiotherapy

The role of radiotherapy in melanoma treatment is still controversial. Literature supports the efficacy of adjuvant and elective irradiation in patients with high-risk clinic-pathologic feature melanoma [61], but the effect on overall survival remains largely unknown.

Whole-brain radiotherapy may provide palliative benefits in patients with multiple brain metastases [62].

Cutaneous SCC is radiosensitive, and several different radiotherapy methods are used, including external beam, low-voltage X-rays, and electrons.

In SCC treatment, radiotherapy is usually reserved for postoperative sterilization of the surgical field after regional dissection for lymph node metastasis.

Retrospective studies suggest that adjuvant radiotherapy improves locoregional control and survival when the staging is higher than N1 or extracapsular tumor spreads [63].

Another indication is the combined treatment of locally advanced SCC with perineural invasion, frequently associated with reduced local control and survival [64].

For BCC, the indication for radiotherapy is strictly confined to advanced inoperable cancers or for patients who will not sustain the surgery, but may benefit from palliative local control of disease [65].

## 8. Conclusions

Skin tumors may be not “appealing” for genetic researchers with respect to gastrointestinal or lung or breast cancer, but their impact on the patient's quality of life can be tremendous, especially when skin cancer starts occurring during childhood without ever ending.

Patients and their families experience the tragedy of a sunless life, since the major triggers for skin cancer development are UV rays.

Despite efforts made to avoid sun exposure with clothes and sunscreens, from adolescence on melanoma and NMSC have great chance to occur on all the body surfaces, even if the head and neck area and dorsum of the hands remain the more subjected to disease.

The texture, color, and softness of the skin suffer progressive alteration, and the healing subsequent to oncologic surgery may be unpleasant and unsatisfying.

For the aforementioned reasons, it would be useful to indicate preventive measures to all the newborns XP diagnosed, in particular to whom present the complementation group alteration more susceptible to skin cancer. Since, in many cases, the high number of lesions in the same area makes conventional resections impractical. Pharmacological and systemic therapy finds wide applications.

Topically, PDT is widely applied for premalignant and malignant lesions of NMSCs. Other drug therapies such as imiquimod alone and fluorouracil alone find application in patients with tumors not candidates for standard ablative treatment.

At the systemic level, target therapy has become an appropriate first-line adjuvant treatment for patients with advanced mutant cutaneous melanoma. Chemotherapy agents have been successfully tested, but due to systemic side effects, their use is limited to second-line treatments. The role of CPI in the treatment of SCC in patients with XP is not clear, but isolated cases suggest the efficacy of cemiplimab for the high mutational burden of tumors.

Hedgehog pathway inhibitors are used in the treatment of patients with BCC even if relapses are frequent upon discontinuation of the drug, which however respond in 50% of cases to a second drug administration.

Far from obtaining conclusive results, this analysis has some strengths.

First, this is a systematic review specifically focused on correlation between XP and skin cancers that was never been conducted before.

Second, the quality of eligible studies included in the current review was satisfactory and met our inclusion criteria.

Third, we succeeded in pointing out the lack of studies and research in the literature on this field, taking into account that many meta-analyses report the same studies, now dated, and characterized by similar publication bias.

Undoubtedly, the absence of a statistical analysis of the stratified sources to obtain adjusted estimates and the small sample of investigations analyzed represent the limits of this study.

## Figures and Tables

**Table 1:** Summary of findings and grade of evidence rating.

Studies selected						Quality assessment						
First author, year	XP complementation group	Gene polymorphism	Risk of cancer	No. of studies	Prevalence of studies on skin cancer	Study design	Risk of bias	Imprecision	Inconsistency	Indirectness	Publication bias	Quality of evidence
Zhao, 2018	XPG	rs17655	All	60	13.3% (8/60)	Meta-analysis	Serious^a,b^	Not serious^c^	Not serious^d^	Not serious^a,e^	Not significant^f^	Moderate
Zebian, 2019	XPC	Lys939Gln	Skin and internal	30 (humans), 13 (mice)	20% (6/30 in humans), 53.8% (7/13 in mice)	Nonsystematic review	Very serious^g^	Not serious	Serious^h^	Not serious	Significant	Very low
		Ala499Val										
		Poly insertion/deletion (PAT^+/-^)										
Shi, 2012	XPF	Arg415Gln	All	43	0% (0/23)	Meta-analysis	Serious^a-b^	Not serious	Not serious^i^	Serious	Not significant^f,j^	Low
		Ser835Ser			9% (1/11)							
		Ser662Pro			n.a. (*x*/5)							
		Intron			n.a. (*x*/4)							
Mandal, 2013	XPD	A751C	All	13	0%	Meta-analysis	Very serious^k^	Serious^l^	Not serious^d^	Not serious^e^	Not significant^f,j^	Very low
Liu, 2019	XPF	rs2276466	All	11	0%	Meta-analysis	Serious^a-b,m^	Serious^n^	Not serious	Not serious	Not significant^f,j^	Very low
He, 2014	XPG	Asp1104His	All	66	15% (10/66)	Meta-analysis	Serious^o^	Not serious	Not serious	Not serious	Not significant^f,j^	Moderate
	XPF	Arg415Gln		32	3% (2/66)							
Han, 2017	XPG	rs751402	All	14	0% (0/14)	Meta-analysis	Serious^o^	Serious^n^	Not serious	Not serious	Not significant^f,j^	Moderate
		rs873601		12	0% (0/12)							
		rs2296147		12	0% (0/12)							
Ding, 2011	XPA	-4G>Ars1800975	All	36	11% (4/36)	Meta-analysis	Serious^o^	Serious^n^	Not serious	Not serious	Not significant^f,j^	Low
Natale, 2017	XPB/XPD/XPF XPG+Cockayne syndrome	CSA/ERCC8 or CSB/ERCC6	All	33	15% (5/33)	Systematic review	Very serious^p^	Very serious^n^	Serious^h^	Not serious^e^	Significant	Very low
Jiang, 2015	XPC	Lys939Gln	Melanoma	8	100% (8/8)	Meta-analysis	Very serious^a,b,o^	Serious^l^	Not serious	Not serious	Not significant^j^	Very low

^a^The study concerns about the correlation between cancer and a single XP gene polymorphism. ^b^Most of the reports referred to a single type of cancer. ^c^The optimal information size (OIS) criterion is met, and the 95% CI excludes no effect. ^d^Significant between-study heterogeneity was detected in the overall analysis. ^e^The study population overlaps with a subgroup of clinical patients. ^f^Symmetry of results in the funnel plot with pseudo 95% confidence limits. ^g^Not indicated inclusion/exclusion search criteria. ^h^Heterogeneity of studies not balanced with proper statistics. ^i^Heterogeneity of studies was balanced by using a random-effect model that generated wider CI. ^j^Egger's test suggested there was no publication bias. ^k^Study limited to Indian population. ^l^The meta-analysis is based primarily on unadjusted effect estimates and CIs. ^m^The number of study was relatively small for some cancer types. ^n^Some event estimate comes from small studies. ^o^Nondifferential misclassification bias is possible due to different reference populations. ^p^Search criteria ambiguous, and no exclusion criteria declared.
